# Case report: Implantable cardioverter-defibrillator implantation with optimal medical treatment for lethal ventricular arrhythmia caused by recurrent coronary artery spasm due to tyrosine kinase inhibitors

**DOI:** 10.3389/fcvm.2023.1145075

**Published:** 2023-03-14

**Authors:** Jifu Jin, Guang Xia, Yu Luo, Ying Cai, Ying Huang, Zhiyun Yang, Qinghui Yang, Bing Yang

**Affiliations:** Department of Cardiology, School of Medicine, Shanghai East Hospital, Tongji University, Shanghai, China

**Keywords:** coronary artery spasm, tyrosine kinase inhibitors, ventricular arrhythmia, sudden cardiac death, implantable cardioverter-defibrillator

## Abstract

Coronary artery spasm (CAS) may induce lethal ventricular arrhythmia due to severe and prolonged vessel constriction. Tyrosine kinase inhibitors are associated with the occurrence of CAS. Optimal medical treatment is the first-line therapeutic option for the management of CAS, whereas patients who experienced aborted sudden cardiac death (SCD) may benefit from implantable cardioverter-defibrillator (ICD) implantation. We report a case of a 63-year-old Chinese man receiving tyrosine kinase inhibitor treatment for liver cancer who presented with recurrent chest discomfort and syncope with an elevation of high-sensitivity troponin T. Emergent coronary angiography showed sub-total occlusion of the left anterior descending artery without other signs of CAS. Percutaneous transluminal coronary angioplasty with a drug-coated balloon was performed successfully with the guidance of intravascular ultrasound. After 5 months, the patient returned to the emergency room for chest discomfort and another episode of syncope. The electrocardiogram showed ST-segment elevation in the inferior and V5–V6 leads compared to the previous event. Coronary angiography was repeated immediately and showed significant luminal stenosis at the midportion of the right coronary artery (RCA), whereas, after administration of intracoronary nitroglycerine, a remarkable recovery of RCA patency was noticed. A diagnosis of CAS was made, and soon after that, the patient rapidly developed ventricular arrhythmia in the coronary care unit. After successful resuscitation, the patient recovered completely and received long-acting calcium channel blockers as well as nitrates therapy. ICD implantation was performed considering the high risk of recurrence of life-threatening ventricular arrhythmia. During the follow-up period, the patient has been free of angina, syncope, or ventricular arrhythmia, and ICD interrogation showed no ventricular tachycardia or ventricular fibrillation. We first reported the case of a patient with CAS induced by regorafenib treatment complicated with severe atherosclerotic coronary disease who survived from sudden cardiac arrest. ICD implantation is indicated in patients who experienced aborted SCD for the prevention of the next lethal ventricular arrhythmia.

## Introduction

Coronary artery spasm (CAS) is a life-threatening syndrome that is characterized by exaggerated vasoconstriction without obstructive coronary artery, which leads to myocardial infarction (1). The clinical presentation of CAS varies from stable angina, acute myocardial infarction, and malignant arrhythmia, to sudden cardiac death (SCD) (2). Currently, CAS is recognized as a multifactorial disease and its underlying pathophysiology is complex and not fully understood. A recent study reported that tumor-targeted therapy may induce coronary spastic events (3). Optimal medical therapy (OMT), including long-acting calcium channel blockers and nitrate, is the baseline treatment for the prevention of CAS recurrence. Nevertheless, an implantable cardioverter-defibrillator (ICD) is recommended for those who have experienced aborted SCD in addition to OMT (4, 5). In this study, we report a case of a patient who had multiple precipitated risk factors for CAS further managed with OMT and simultaneous ICD implantation for secondary prevention of SCD.

## Case description

**Figure 1:**
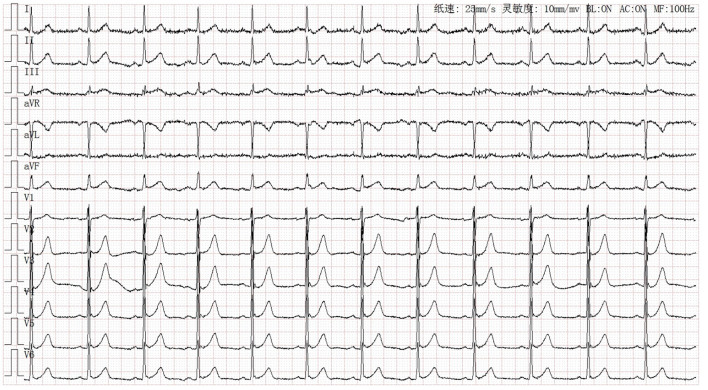
Electrocardiogram at the first admission showed sinus rhythm and early repolarization pattern with a J-wave in the inferior and V5–V6 leads.

**Figure 2:**
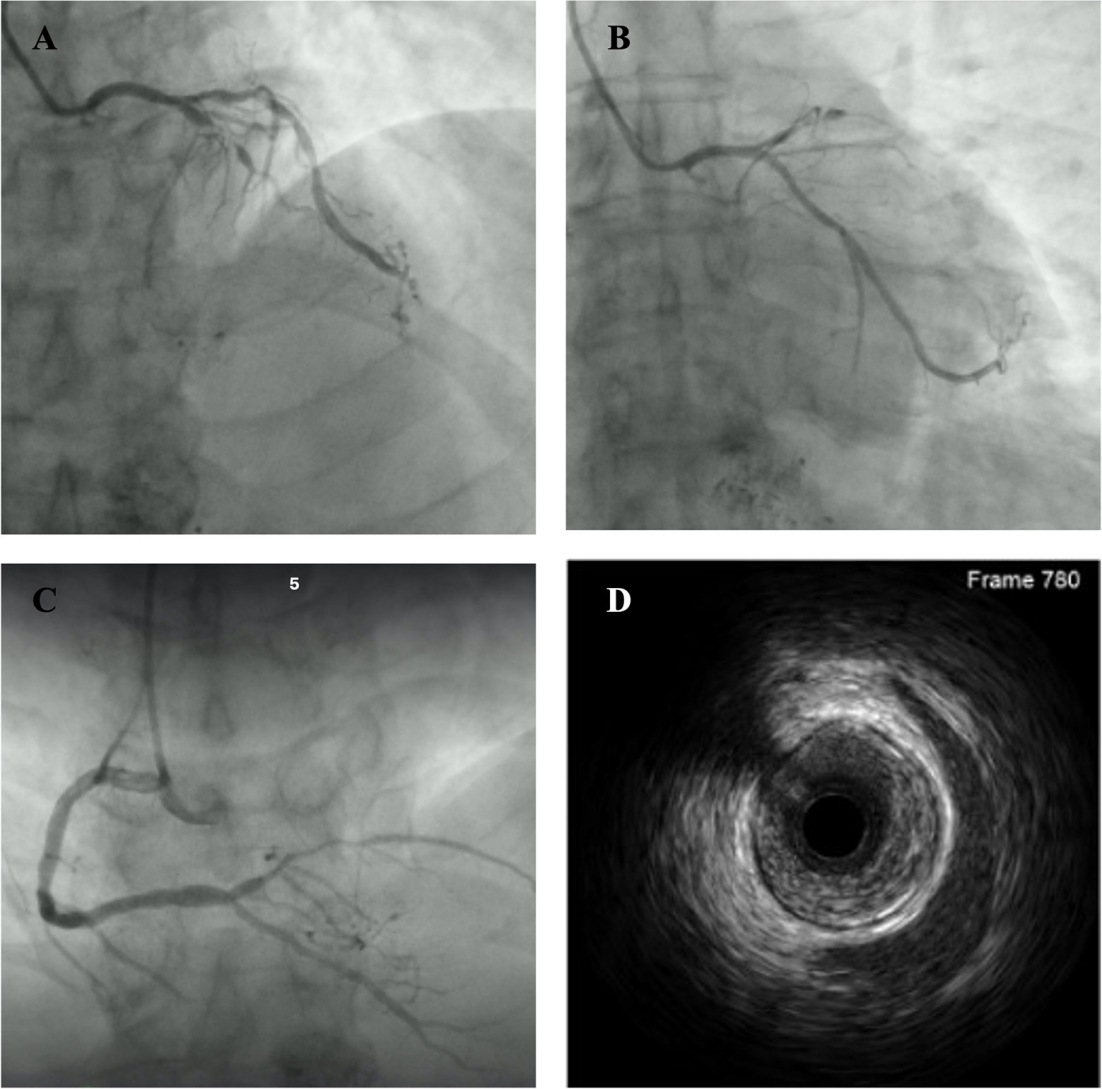
Baseline coronary angiography at first admission showed a subtotal occlusion of the left anterior descending artery with thrombolysis in myocardial infarction 0–1 flow **(A)**, mild–moderate stenosis in the left circumflex artery **(B)**, and right coronary artery **(C)**. Intravascular ultrasound showed diffused atherosclerotic plaque in the left anterior descending artery **(D)**.

A 63-year-old Chinese man was referred to our institution due to transient chest pain followed by one episode of syncope for several minutes. He had hypertension, diabetes mellitus, and former smoking as cardiovascular risk factors. He had no prior history of syncope or a family history of juvenile SCD. He received a hepatectomy for liver cancer in 2017 and a lobectomy for lung cancer in 2018. He was treated with transarterial chemoembolization and regorafenib as adjunctive therapies for liver cancer. At the presentation to the emergency room, he appeared comfortable with unlabored breathing. He had a blood pressure of 167/73 mmHg, heart rate of 73 b.p.m., and oxygen saturation of 100% on room air. High-sensitivity troponin T level was increased to 0.399 ng/ml (reference range <0.014 ng/ml). An electrocardiogram (ECG) showed sinus rhythm and signs of early repolarization pattern with a J-wave in the inferior and V5–V6 leads (Figure 1). He was diagnosed with acute coronary syndrome, and cardiac catheterization was performed immediately. Coronary angiography (CAG) revealed a subtotal occlusion of the proximal left anterior descending (LAD) artery with antegrade thrombolysis in myocardial infarction (TIMI) 0–1 flow (Figure 2A). CAG also indicated mild–moderate stenosis in the left circumflex artery (LCX) and right coronary artery (RCA) with TIMI 3 flow (Figures 2B, C). The mid to distal LAD vessel was filled from collaterals originating from the distal RCA. Intravascular ultrasound (IVUS) was performed in LAD after balloon pre-dilation and it identified diffused atherosclerotic plaque from proximal to distal LAD (Figure 2D). After sufficient pretreatment with a cutting balloon and non-compliant balloon, we performed percutaneous transluminal coronary angioplasty (PTCA) with the drug-coated balloon (RESTORE, 3.0 × 15 mm, CARDIONOVUM, GERMANY) in the culprit lesion of LAD. The patient was discharged successfully with a prescription of aspirin 100 mg daily, ticagrelor 90 mg twice daily, rosuvastatin 10 mg daily, benazepril 2.5 mg daily, and bisoprolol 2.5 mg daily until a clinical follow-up.

**Figure 3:**
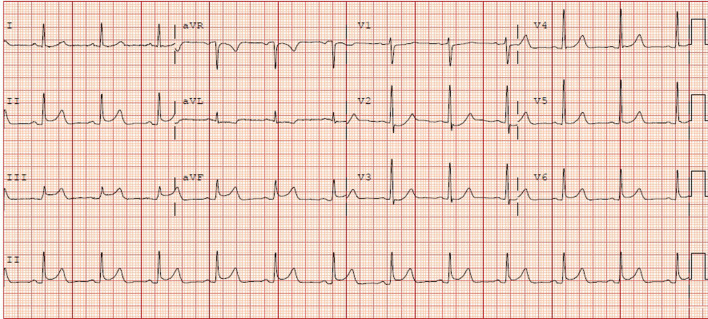
Electrocardiogram recorded on the second admission showed sinus rhythm, ST-segment elevation in the inferior and V5–V6 leads, and ST-segment depression in the lateral leads.

**Figure 4:**
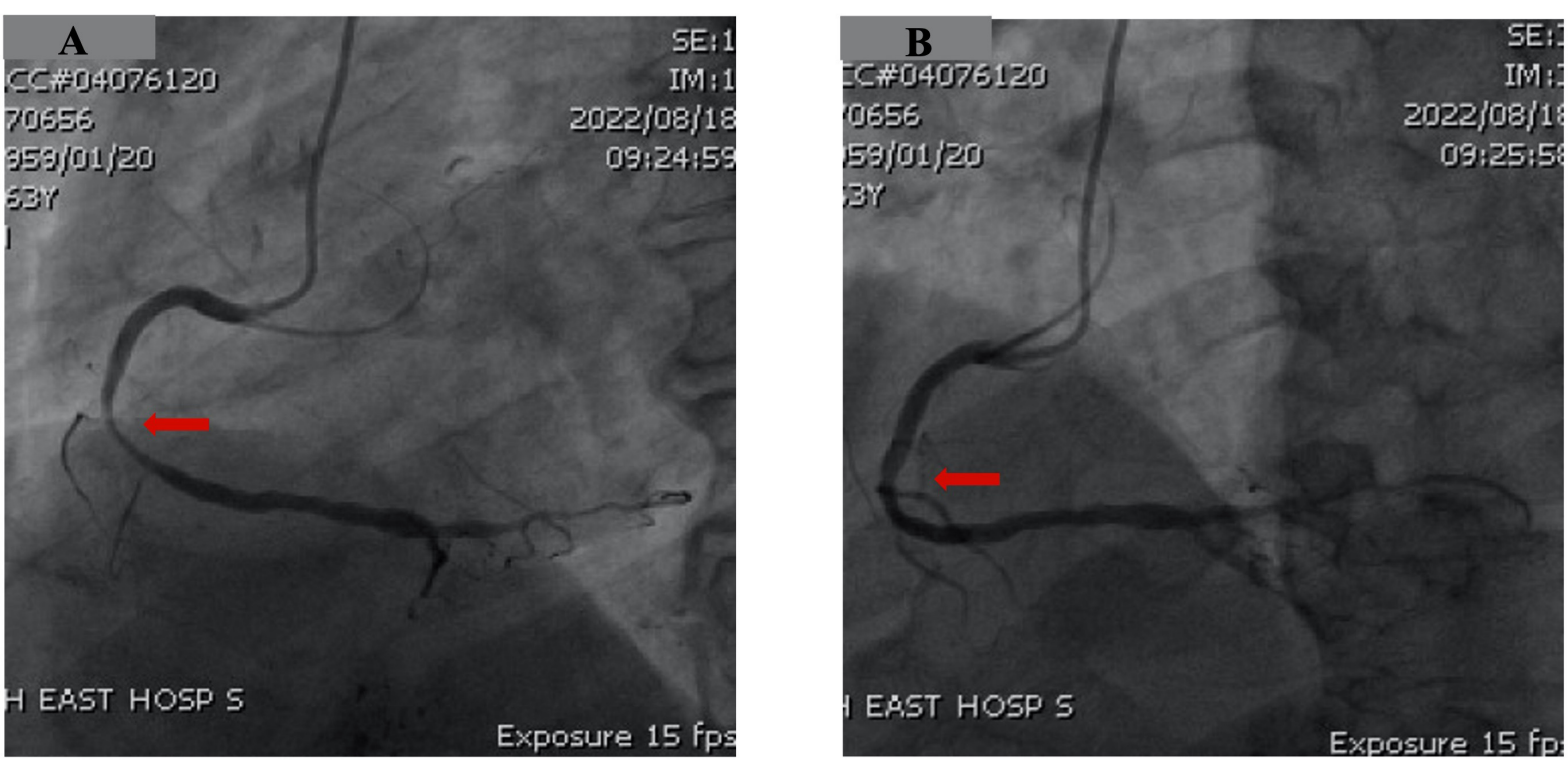
Follow-up coronary angiography showed severe stenosis of the right coronary artery (**A**, red arrow). After administration of intracoronary nitrates, a significant recovery of vessel patency was observed **(B)**, and a diagnosis of epicardial spasm was made (**B**, red arrow).

Nevertheless, 5 months later, the patient came back to our emergency room for recurrent chest discomfort, dyspnea, and another episode of syncope that occurred 6 h before. Physical examination, high-sensitivity troponin T, D-dimer, and head computed tomography were normal; however, ECG indicated ST-segment elevation in the inferior and V5–V6 leads when compared to the previous event (Figure 3). CAG showed significant luminal stenosis at mid-RCA; nevertheless, lesions in LAD and LCX were approximately unchanged when compared to the previous examination (Figure 4A). After the administration of intracoronary nitroglycerine, a remarkable recovery of RCA patency was noticed, and a diagnosis of coronary artery spasm was illustrated (Figure 4B). Bisoprolol was discontinued, and oral administration of nitroglycerine and calcium channel blockers was initiated for the prevention of CAS. Unfortunately, he suddenly developed unconsciousness and hemodynamic collapse soon after being back in the coronary care unit. Dynamic ECG records showed severe sinus bradycardia, advanced atrioventricular block, ventricular escape rhythm, and ventricular fibrillation (Supplementary Figure 1). After successful resuscitation with chest compression, intubation, and defibrillation, his neurological status gradually improved with a Glasgow coma score of 15/15. After the acute phase passed, his ECG and left ventricular contraction were completely normal. Intravenous injections of nitroglycerine and diltiazem were administrated as well. His ECG monitor indicated no ventricular arrhythmia during hospitalization. Taking all these findings together, we attributed the patient's symptoms, namely, angina, syncope, and ventricular arrhythmia, to coronary artery spasm and implanted an ICD for secondary prevention of SCD (Supplementary Figure 2). He was discharged on diltiazem and isosorbide mononitrate as well as aspirin and rosuvastatin. During the 3 months follow-up, the patient had been free of chest discomfort, dyspnea, or syncope, and ICD interrogation showed no ventricular tachycardia or ventricular fibrillation.

## Discussion

Coronary artery spasm is regarded as a detrimental disorder that may induce lethal arrhythmia or sudden cardiac arrest due to severe constriction of the coronary artery. In addition to the traditional risk factors, such as age, sex, and smoking, previous studies reported that targeted therapy for cancer was associated with the occurrence of CAS (3, 6). A recent study described that tyrosine kinase inhibitors (TKIs) were related to CAS possibly through the upregulation of the Rho/ROCK pathway and augmentation of Ca^2+^ sensitization resulting in hyper-constriction of coronary smooth muscle (6). Fiets et al. reported two chronic myeloid leukemia cases with CAS likely due to multiple TKI usage, including nilotinib, dasatinib, and bosutinib (3). In this case, to our knowledge, we first presented that regorafenib, as one of the TKIs, might be a contributing risk factor for CAS along with known risk factors, such as hypertension, diabetes mellitus, and hyperlipidemia. Considering that regorafenib was related to the CAS occurrence, we recommended ceasing the suspected drugs; however, the patient insisted on TKI treatment for advanced liver cancer.

Although CAS is regarded as a functional disease of the coronary artery, it may also occur with severe coronary artery stenosis. The prevalence of coexisting CAS and significant atherosclerotic stenosis was within 10% of what was reported in a previous Japanese study (7). Takagi et al. proposed that significant coronary artery atherosclerotic stenosis independently increased the risk of cardiovascular events in patients with CAS who survived SCD (7). Recently, a prospective multicenter study also indicated that significant coronary artery stenosis was strongly associated with 1-year ACS event and composite adverse clinical outcomes in patients with CAS (8). In our case, the patient presented significant atherosclerotic stenosis combined with recurrent coronary spasms characterized by syncope, ACS, and lethal arrhythmia. Optimal management of traditional cardiovascular risk factors and the prevention of vasospasm are both of great importance to improve a patient's long-term outcome.

Appropriate medical treatment for coronary spasms consists of long-acting calcium channel blockers, long-acting nitrates and nicorandil, management of reversible risk factors, and cessation of beta-blockers if possible (9, 10). A previous study indicated that the Rho-kinase inhibitor fasudil could be a beneficial treatment for CAS (11). A recent case report also described an off-label use of riociguat, a soluble guanylate cyclase stimulator, for the treatment of refractory angina from coronary spasm despite conventional antianginal treatment (12). Studies have shown that optimal medical treatment may improve the severity of variant angina, whereas patients who experienced aborted SCD due to CAS may have a poor long-term prognosis (13–15). In such cases, ICD implantation is recommended in CAS patients with aborted SCD for the prevention of the next life-threatening fatal arrhythmia (4). Sueda and Kohno reported that appropriate ICD shocks were observed in 24.1% of CAS patients with aborted SCD who received ICD implantation in the first event (15). European Society of Cardiology (ESC) guidelines for the management of ventricular arrhythmia for the prevention of SCD (16) updated the recommendation of ICD implantation in cardiac arrest survivors with coronary spasm as class IIa when considering that patients were at a high risk of a next fatal ventricular arrhythmia. According to the recurrent episodes of syncope, fatal arrhythmia, and inability to discontinue TKI treatment, we believe that the implantation of ICD combined with OMT is an appropriate therapeutic strategy for our patient.

In summary, we first reported a case of a patient with coronary artery spasm induced by regorafenib treatment complicated with severe atherosclerotic coronary disease who survived sudden cardiac arrest. ICD implantation is indicated in patients who experienced aborted SCD for the prevention of the next lethal ventricular arrhythmia.

## Data availability statement

The raw data supporting the conclusions of this article will be made available by the authors, without undue reservation.

## Ethics statement

The studies involving human participants were reviewed and approved by Institutional Review Board of Shanghai East Hospital. The patients/participants provided their written informed consent to participate in this study.

## Author contributions

JJ and GX wrote the manuscript. ZY collected the data. YL, YC, YH, QY, and BY guided the whole process of the presentation. All authors contributed to the article and approved the submitted version.

## Supplementary material

Supplementary Figure 1 Sequential electrocardiogram records after being back in the coronary artery unit showing atrioventricular block, ventricular escape rhythm **(A)**, and successful resuscitation **(B)**.

Supplementary Figure 2 Chest X-ray after implantation of the implantable cardioverter-defibrillator.

Supplementary Figure 3 Timeline of events during the hospital stay. LAD, left anterior descending; CAS, coronary artery spasm; CCU, coronary care unit; and ICD, implantable cardioverter-defibrillator.
