# Supplementary figures and images for: Case report: Implantable cardioverter-defibrillator implantation with optimal medical treatment for lethal ventricular arrhythmia caused by recurrent coronary artery spasm due to tyrosine kinase inhibitors
Source: Front Cardiovasc Med. 2023 Mar 14;10:1145075. doi: 10.3389/fcvm.2023.1145075 (PMC10045981; doi:10.3389/fcvm.2023.1145075)

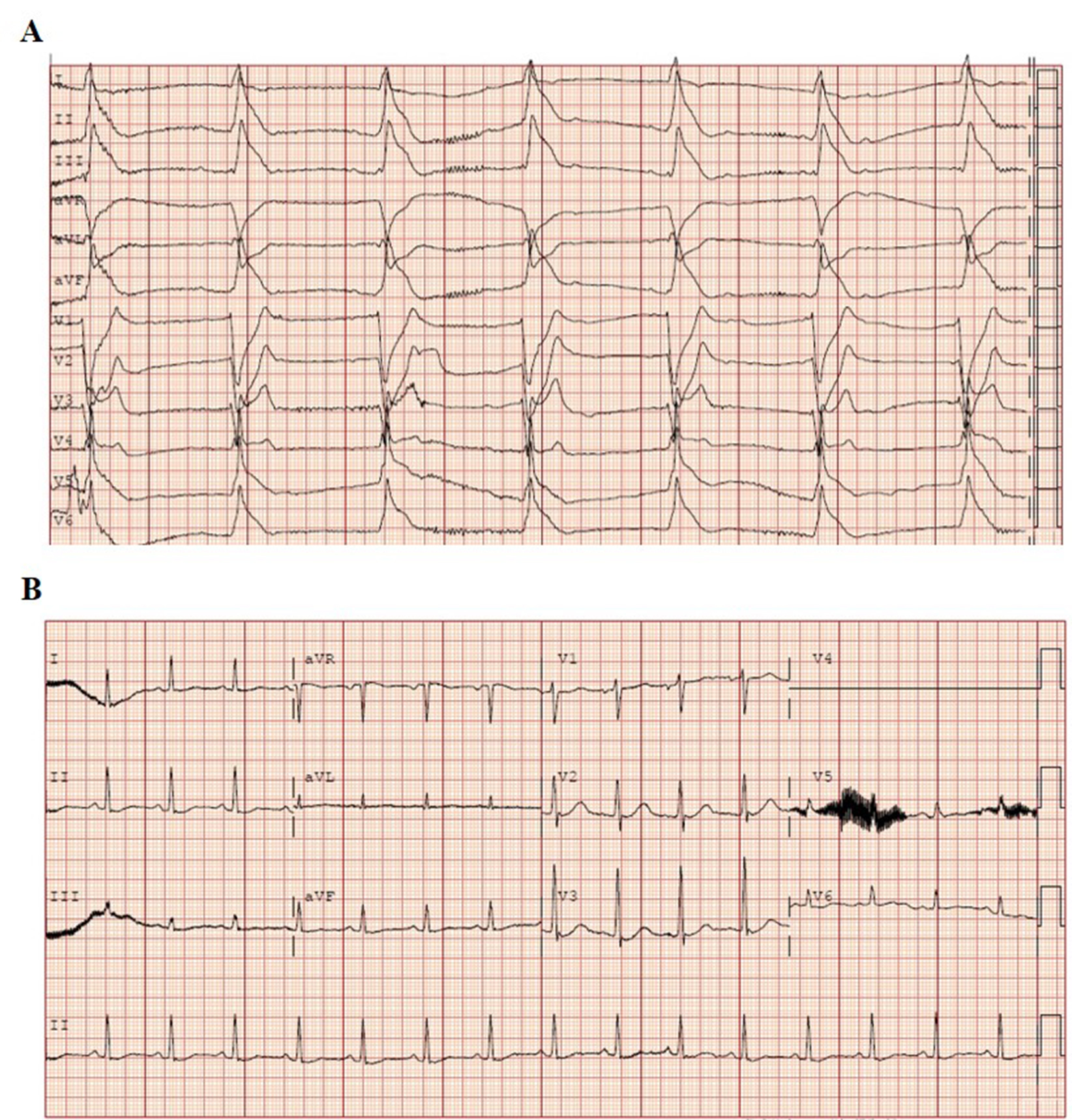

Supplement: Supplementary Figure 1 — Sequential electrocardiogram records after being back in the coronary artery unit showing atrioventricular block, ventricular escape rhythm (A), and successful resuscitation (B). [file Image_1.JPEG]

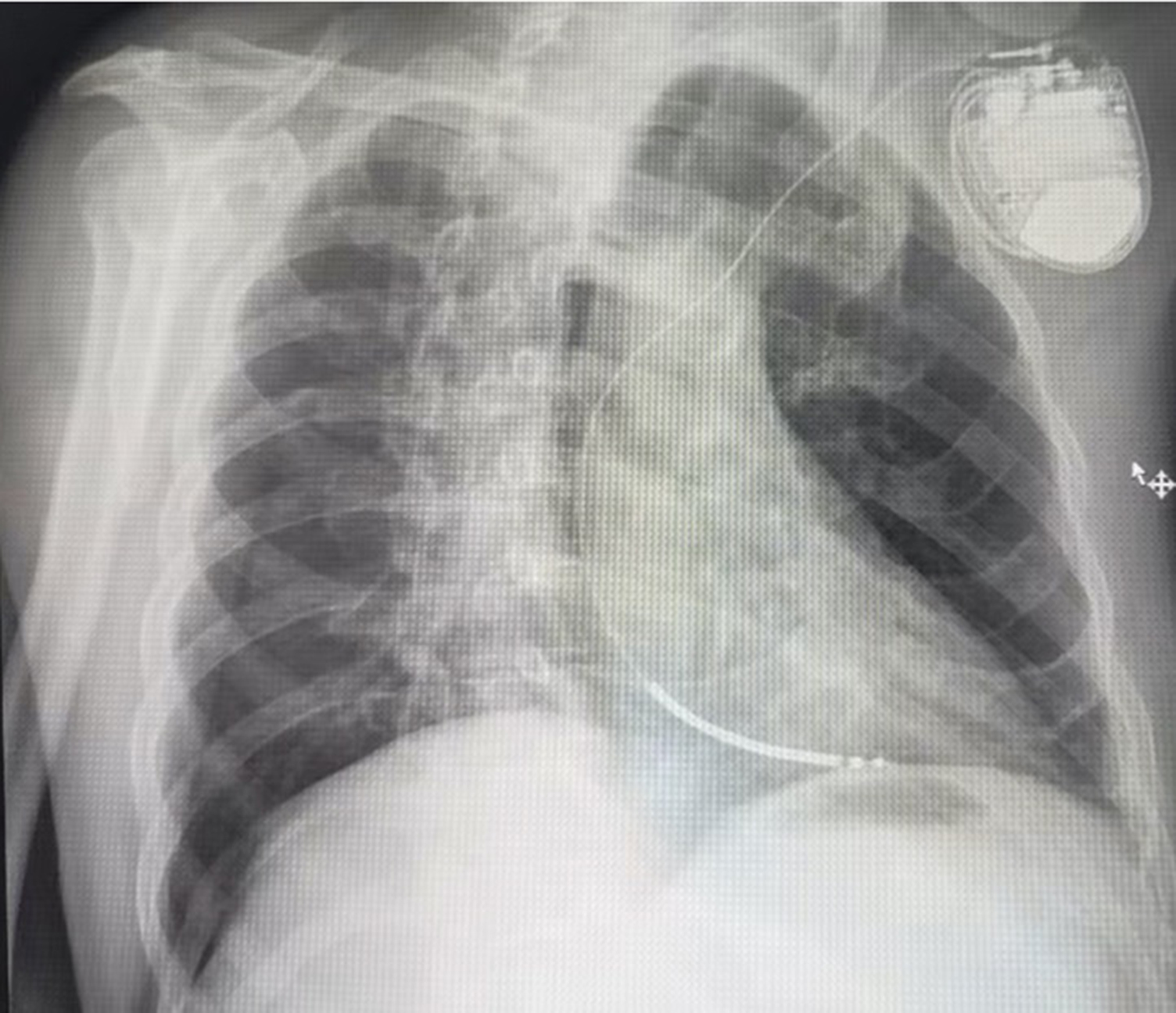

Supplement: Supplementary Figure 2 — Chest X-ray after implantation of the implantable cardioverter-defibrillator. [file Image_2.JPEG]

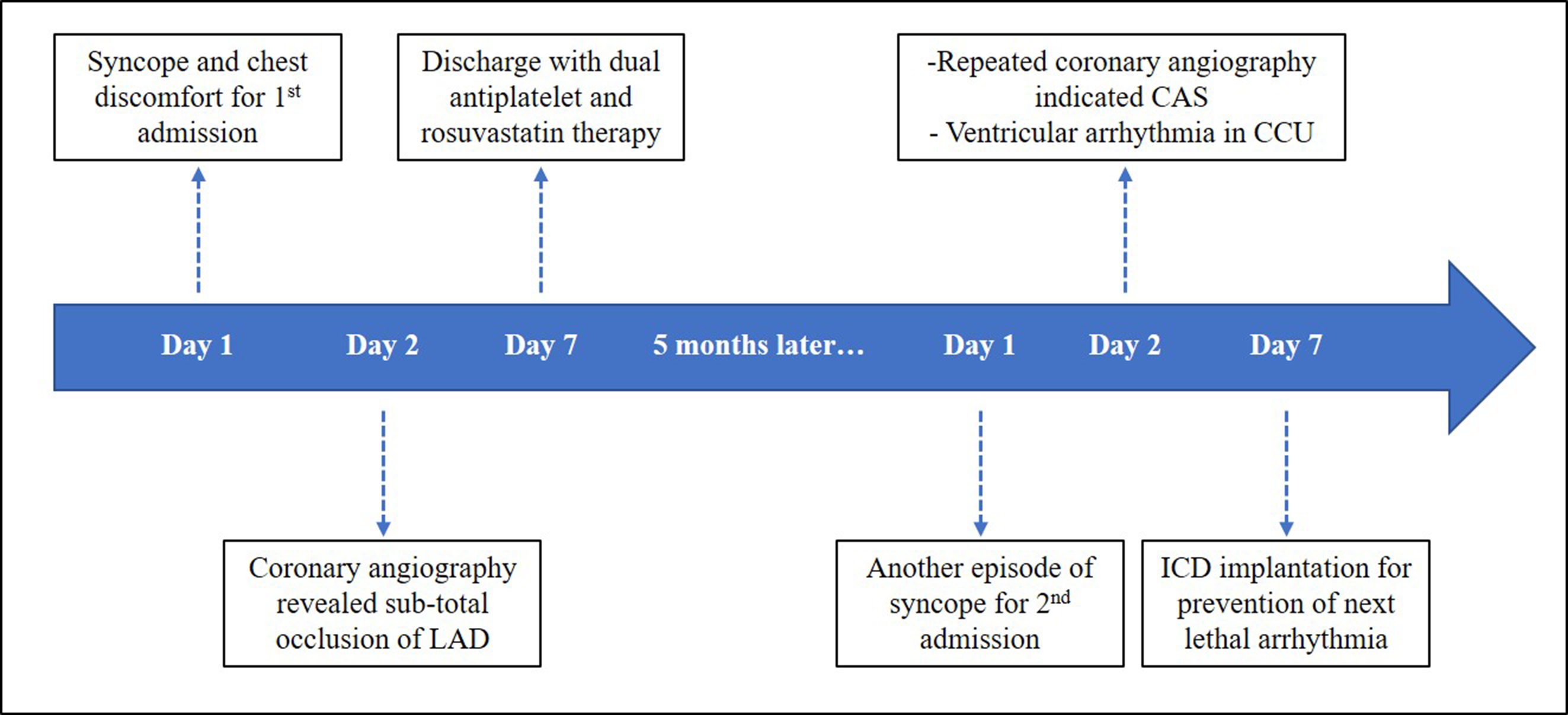

Supplement: Supplementary Figure 3 — Timeline of events during the hospital stay. LAD, left anterior descending; CAS, coronary artery spasm; CCU, coronary care unit; and ICD, implantable cardioverter-defibrillator. [file Image_3.JPEG]
